# Osteoclast-like stromal giant cells in breast cancer likely belong to the spectrum of immunosuppressive tumor-associated macrophages

**DOI:** 10.3389/fmolb.2022.894247

**Published:** 2022-08-26

**Authors:** Elham Sajjadi, Gabriella Gaudioso, Andrea Terrasi, Francesca Boggio, Konstantinos Venetis, Mariia Ivanova, Letizia Bertolasi, Gianluca Lopez, Letterio Runza, Alice Premoli, Daniele Lorenzini, Elena Guerini-Rocco, Stefano Ferrero, Valentina Vaira, Nicola Fusco

**Affiliations:** ^1^ Division of Pathology, IEO, European Institute of Oncology IRCCS, Milan, Italy; ^2^ Department of Oncology and Hemato-Oncology, University of Milan, Milan, Italy; ^3^ Division of Pathology, Fondazione IRCCS Ca’ Granda—Ospedale Maggiore Policlinico, Milan, Italy; ^4^ Division of Molecular Biology, Biomedical Center, Faculty of Medicine, LMU Munich, Planegg-Martinsried, Munich, Germany; ^5^ Department of Biomedical, Surgical, and Dental Sciences, University of Milan, Milan, Italy; ^6^ Department of Pathophysiology and Transplantation, University of Milan, Milan, Italy

**Keywords:** breast cancer, osteoclast-like giant cells (OGCs), osteoclast-like stromal giant cells, tumor microenevironment, tumor immune microenvironment, tumor-infiltrating lymphocytes (TILs), tumor-associated macrophages (TAMs), miRNA

## Abstract

**Background:** Breast cancer with osteoclast-like stromal giant cells (OSGC) is an exceedingly rare morphological pattern of invasive breast carcinoma. The tumor immune microenvironment (TIME) of these tumors is populated by OSGC, which resemble osteoclasts and show a histiocytic-like immunophenotype. Their role in breast cancer is unknown. The osteoclast maturation in the bone is regulated by the expression of cytokines that are also present in the TIME of tumors and in breast cancer tumor-associated macrophages (TAMs). TAMs-mediated anti-tumor immune pathways are regulated by miRNAs akin to osteoclast homeostasis. Here, we sought to characterize the different cellular compartments of breast cancers with OSGC and investigate the similarities of OSGC with tumor and TIME in terms of morphology, protein, and miRNA expression, specifically emphasizing on monocytic signatures.

**Methods and Results:** Six breast cancers with OSGC were included. Tumor-infiltrating lymphocytes (TILs) and TAMs were separately quantified. The different cellular populations (i.e., normal epithelium, cancer cells, and OSGC) were isolated from tissue sections by laser-assisted microdissection. After RNA purification, 752 miRNAs were analyzed using a TaqMan Advanced miRNA Low-Density Array for all samples. Differentially expressed miRNAs were identified by computing the fold change (log2Ratio) using the Kolmogorov-Smirnov test and *p* values were corrected for multiple comparisons using the false discovery rate (FDR) approach. As a similarity analysis among samples, we used the Pearson test. The association between pairs of variables was investigated using Fisher exact test. Classical and non-classical monocyte miRNA signatures were finally applied. All OSGC displayed CD68 expression, TILs (range, 45–85%) and high TAMs (range, 35–75%). Regarding the global miRNAs profile, OSGC was more similar to cancer cells than to non-neoplastic ones. Shared deregulation of miR-143-3p, miR-195-5p, miR-181a-5p, and miR-181b-5p was observed between OSGC and cancer cells. The monocyte-associated miR-29a-3p and miR-21-3p were dysregulated in OSGCs compared with non-neoplastic or breast cancer tissues.

**Conclusion:** Breast cancers with OSGC have an activated TIME. Shared epigenetic events occur during the ontogenesis of breast cancer cells and OSGC but the innumophenotype and miRNA profiles of the different cellular compartmens suggest that OSGC likely belong to the spectrum of M2 TAMs.

## Introduction

Breast cancer with osteoclast-like stromal giant cells (OSGC) is a rare tumor showing a variable number of OSGC at the periphery of the neoplastic nests and/or within the tubular lumens, in the context of a hypervascular microenvironment composed of lymphocytes, histiocytes, and monocytes (Zhou et al., 2014; Marchiò et al., 2015; Peña-Jaimes et al., 2018; WHO Classification of Tumours Editorial Board and WHO Classification of Breast Tumours, 2019). As their name suggests, OSGC resemble osteoclasts (Ohashi et al., 2018a; Caetano Oliveira and Schmitt, 2018; Bonsang et al., 2019; Hoda et al., 2020). They are morphologically characterized by an abundant intensely-eosinophilic cytoplasm containing well-developed organelles and numerous oval non-atypical nuclei with prominent nucleoli (Ellis et al., 2020; Behzatoglu, 2021). By immunohistochemical analysis, OSGC show the expression of histiocytic surface markers (e.g., CD68) and are negative for cytokeratins, S100, actin, and markers of cell proliferation such as Ki67 (Ohashi et al., 2018b; Ofri et al., 2020). There is still no sufficient clinical evidence available on the value of reporting the OSGC presence and amount in breast neoplasms. For this reason, breast cancers with OSGC are classified as a morphological pattern of invasive breast carcinoma of no special type (NST) and not as a special histological type (WHO Classification of Tumours Editorial Board and WHO Classification of Breast Tumours, 2019).

Regarding the nature of OSGC in breast cancer, there is a general agreement on their histiocytic origin, as proposed for other tumor types with an OSGC component (Bonsang et al., 2019; Xu et al., 2019; Liu et al., 2021; Mori et al., 2021). Due to their multinucleated appearance and global ultrastructure, some authors have posited that OSGC are generated by a syncytial fusion of macrophages, similar to what happens to osteoclasts during osteoclastogenesis (Shishido-Hara et al., 2010; Liu et al., 2018; Liang et al., 2019; Invernizzi et al., 2020a; Inoue et al., 2021; Kao et al., 2021; Venetis et al., 2021). Researchers have been looking for further parallels between OSGC and osteoclasts, whose formation is regulated by the expression of cytokines, including tumor necrosis factor-α (TNFα), interleukin-1α (IL1α), macrophage-colony stimulating factor (M-CSF), and receptor activator of NF-κB ligand (RANKL) (Kolb et al., 2019; Invernizzi et al., 2020b; Liang et al., 2021). These molecules have been documented both in the tumor immune microenvironment (TIME) of other neoplasms with an OSGC component (Bennàssar et al., 2011; Hatano et al., 2014; Fusco et al., 2017a; Liang et al., 2021) and in breast cancer tumor-associated macrophages (TAMs) (Lala et al., 2018; Guo et al., 2020). In breast cancer, the presence of tumor-infiltrating lymphocytes (TILs) and TAMs within the TIME are related to the secretion of specific cytokines that are involved in immunosuppression, angiogenesis, tumor progression, and metastasis (Mao et al., 2013; Pagni et al., 2019; Horimoto et al., 2020; Criscitiello et al., 2021; Fusco et al., 2022). It has become increasingly evident that miRNAs play a crucial role in regulating TAMs-mediated anti-tumor immune pathways as well as in osteoclast differentiation, function, and survival (Inoue et al., 2021; O'Brien et al., 2018; McGuire et al., 2015; Fumagalli et al., 2017; Weivoda et al., 2021). However, their role in breast cancer with OSGC is essentially unexplored and based on a handful of morphology-based case reports available in the literature.

We hypothesized that OSGC share not only phenotypic but also molecular features with macrophages/monocytes populating breast cancer TIME. In this study, we sought to characterize the different cellular compartments of breast cancers with OSGC and investigate the similarities of OSGC with tumor and TIME in terms of morphology, protein, and miRNA expression, specifically emphasizing on monocytic signatures.

## Materials and methods

### Patients and tissue specimens

This study is in line with the local ethical guidelines and was approved by the Institutional Review Board (IRB) of Fondazione IRCCS Ca’ Granda - Ospedale Maggiore Policlinico under the protocol number # 620-2018bis. Only therapy-naïve patients and their corresponding surgical specimens were included in this study. Taken together, 6 cases of breast cancers with OSGC were retrieved from the pathology archives of the aforementioned Institution. Hematoxylin and eosin-stained serial sections of each case were centrally reviewed, re-classified, and re-graded by two pathologists (F.B. and N.F.), according to the latest WHO recommendations (WHO Classification of Tumours Editorial Board and WHO Classification of Breast Tumours, 2019) and the Nottingham histologic grading system (Rakha et al., 2008), respectively. Pathologic re-staging was performed following the 8th edition of the American Joint Committee on Cancer (AJCC) Cancer Staging Manual (Amin et al., 2017).

### Immunohistochemical analysis

Representative 4-μm-thick sections of all cases were subjected to immunohistochemical analysis (IHC) with antibodies against estrogen receptor (ER), progesterone receptor (PgR), Ki67, HER2, CD68, Tartrate-resistant acid phosphatase (TRAP), and receptor activator of nuclear factor κ B (RANK), as previously described (Fusco et al., 2018; Lopez et al., 2020). Briefly, the protocol uses the Dako automated staining platform (Dako Omnis; Dako, Carpinteria, CA, United States) and anti-human prediluted antibodies. For each antibody, positive and negative controls were included in each slide run. ER, PgR, and HER2 status were tested and reported according to the latest breast biomarker reporting guidelines published by the College of American Pathologists (CAP) (Allison et al., 2020; Wei et al., 2021). The proliferation index was assessed by Ki67 IHC as the global (average) score across the section. According to the updated recommendations from the International Ki67 in Breast Cancer Working Group, a cut-off value of ≥30% was used to define the high proliferation group (Nielsen et al., 2020). Cut-off point for CD68, TRAP, and RANK was calculated as the number of immunoreactive cells (membrane and/or cytoplasm staining of any intensity) divided into lower and higher groups according to the median of a larger cohort (Kuroda et al., 2021). Based on this assumption, a cut-off value of ≥ 26 identified high expressors. The methods and scoring systems employed are detailed in Table 1.

**TABLE 1:** List of antibodies, clones, dilutions, antigen retrieval methods, and scoring systems adopted for immunohistochemical analyses.

Marker	Clone	Dilution	Technology	Antigen retrieval	Scoring
ER	EP1	Ready to use	Dako Omnis	EnVision FLEX, High pH, 20′	ASCO/CAP and St Gallen guidelines; positive if ≥ 1% of tumor cell nuclei are immunoreactive
PgR	PgR 636	1:100	Dako Omnis	EnVision FLEX, High pH, 30′	ASCO/CAP and St Gallen guidelines; positive if ≥ 1% of tumor cell nuclei are immunoreactive
Ki67	MIB1	Ready to use	Dako Omnis	EnVision FLEX, High pH, 30′	International Ki67 in Breast Cancer Working Group; high if ≥ 30% of tumor cell nuclei are immunoreactive
HER2	Polyclonal	1:400	Dako Omnis	EnVision FLEX, Low pH, 30′	ASCO/CAP guidelines; 3 + if complete membrane staining that is intense and >10% of tumor cells; 2 + if weak to moderate complete membrane staining in >10% of tumor cells or complete membrane staining that is intense but within ≤10% of tumor cells; 1 + if incomplete membrane staining that is faint/barely perceptible and within >10% of tumor cells; 0 if no staining observed or membrane stating that is incomplete and is faint/barely perceptible and within ≤10% of tumor cells
CD68	PG-M1	1:100	Dako Omnis	EnVision FLEX, High pH, 30′	Positive for any intensity of membrane/cytoplasm staining; high if ≥ 26 immunoreactive cells
TRAP	sc-59981	1:100	Dako Omnis	EnVision FLEX, High pH, 30′	Positive for any intensity of membrane/cytoplasm staining; high if ≥ 26 immunoreactive cells
RANK	sc-376875	A:100	Dako Omnis	EnVision FLEX, High pH, 30′	Positive for any intensity of membrane/cytoplasm staining; high if ≥ 26 immunoreactive cells

Abbreviation: ER, estrogen receptor alpha; PgR, progesterone receptor; TRAP, tartrate-resistant acid phosphatase; RANK, receptor activator of NFkB.

### Tumor-infiltrating lymphocytes and tumor-associated macrophages assessment

The presence and relative proportion of stromal TILs were determined according to the recommendation of the International TILs Working Group (Dieci et al., 2018). In particular, TILs within the borders of the tumor areas and/or nests were defined as stromal TILs, while the lymphocytes in direct cell-to-cell contact with the tumor cells with no intervening stroma were defined as intratumoral TILs and disregarded from the analysis (Sajjadi et al., 2020; Esposito et al., 2021). The density of stromal TILs was recorded as a continuous percentage and categorized for analyses as negative if 0%, low in between 1 and 10%, intermediate in between 11 and 50%, and high if > 51% (Fusco et al., 2020a). The evaluation and quantification of TAMs were carried out semiquantitatively based on the presence and relative proportion of CD68^+^ mononuclear cells within the TIME per high power field, as previously described (Kuroda et al., 2021).

### Microdissection and RNA extraction

Representative FFPE tissue blocks of 6 patients were selected based on the amount of OSGC; 4-μm-thick sections were then cut and stained with hematoxylin. Subsequently, the histologically distinct components of each case and matched normal breast tissues were separately microdissected using a combination of laser-capture and manual microdissection, as previously described (Fusco et al., 2017b). Specifically, a laser-capture microscope (LMD 6000 System; Leica Biosystems, Wetzlar, Germany) was used for the isolation and microdissection of OSGC. Afterward, the neoplastic epithelial component was isolated from the TIME and manually microdissected using a sterile needle under a stereomicroscope (Zeiss, TIEsse Lab) to ensure > 80% of tumor cell content. Finally, matched normal breast tissue containing non-neoplastic terminal duct-lobular units were manually microdissected. The OSGC (*n* = 6), tumor (*n* = 6), and normal tissue samples (*n* = 6) of each case were collected into separate tubes (*n* = 18) and subjected to total RNA purification using the Master Pure RNA purification kit (Epicenter Biotechnologies, Illumina) as described (Faversani et al., 2021). Next, the RNA content and quality were measured using a spectrophotometer (Thermo Scientific NanoDrop™ 1,000 Spectrophotometer). Samples with poor quantities of miRNA were re-cut, re-microdissected, and re-extracted. All preparation and handling procedures were conducted under RNase-free conditions.

### miRNA profiling

100 ng of total RNA per sample was reverse transcribed using the TaqMan Advanced miRNA cDNA synthesis kit. Then, miRNA profiles were obtained using the TaqMan™ Advanced miRNA Human A and B Cards. A total of 752 miRNAs were assessed (Supplementary Table S1). As a threshold for expression, we set a Cycle threshold (Ct) less than 35. All miRNAs with a Ct value > 35 were considered not expressed. If a miRNA was undetectable (Ct > 35) in the majority plus one sample of our series, it was excluded from further analysis. According to this criterion, 130 miRNAs were available for the study (Supplementary Table S2).

### Statistical and bioinformatics analysis

For miRNA analysis, miRNA raw data were normalized using the most stable miRNAs. As normalizator, miRNAs with a mean < 32 Ct and standard deviation < 2.5 were selected. Then, miRNA relative quantities were median-normalized, log2-transformed, and imported in R environment for statistical analysis. Corrplot packages was used for correlation analysis (https://github.com/taiyun/corrplot). Supervised clustering analysis was performed using the ComplexHeatmap package available within Bioconductor (https://bioconductor.org/packages/release/bioc/html/ComplexHeatmap.html), as previously described (Gu et al., 2016; Faversani et al., 2021). Differentially expressed miRNAs according to clinical or outcome variables were identified by computing the fold change (log2Ratio) between the 2 classes and applying the Wilcox test. The accuracy of the data was assessed using *p* value the false discovery rate (FDR) approach. The associations between pairs of variables were investigated using Fisher exact test (MedCalc software). Differences among samples were analyzed using a non-parametric two-sided Student’s t-test, or Wilcoxon signed-rank test. Statistical analyses were performed using GraphPad Prism version 4 for Windows. A *p* < 0.05 was considered statistically significant.

## Results

### Clinicopathological features and tumor immune microenvironment composition

**FIGURE 1:**
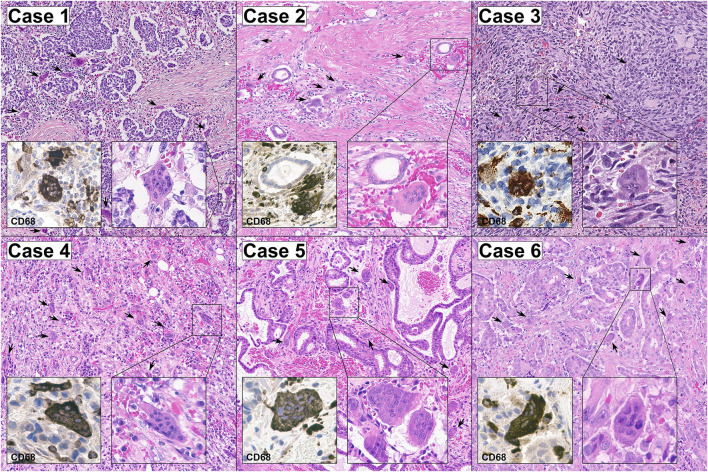
Representative micrographs of the six breast cancers with OSGC included in this study. The stroma of all cases was hypervascular/hemorrhagic and populated by OSGC (arrows) within an activated tumor immune microenvironment characterized by the presence of tumor-infiltrating lymphocytes (TILs) with a relatively high proportion of tumor-associated macrophages (TAMs). The OSGC were characterized by an abundant eosinophilic cytoplasm with several non-atypical nuclei with evident nucleoli. At immunohistochemical analysis, both OSGC and TAMs were CD-68 positive. Original magnification ×100; insets 400x.

All patients (*n* = 6, age range, 35–69; mean ± SD, 56.8 ± 8.8) were diagnosed with intermediate/high grade, highly proliferative (Ki67 ≥ 30) breast cancer. Lymphovascular invasion was observed in 2 (33.3%) cases. All OSGC within each case displayed high CD68 and TRAP expression, and low/null RANK positivity (Supplementary Figure S1), supporting their monocytic origin. In addition to OSGC, in all tumors, both the presence of stromal TILs (range, 45–85%) and the presence of high intratumoral and stromal TAMs (range, 35–75%) were observed. The clinicopathologic characteristics and subtypes of the patients included in this study are listed in Table 2 and shown in Figure 1.

**TABLE 2:** Clinicopathological features of breast carcinomas with OSGC.

Case	Age	Histology	ER	PgR	Ki67	HER2	Grade	LVI	TILs (%)	TAMs (%)	T	N	Stage
OSGC1	69	NST	Neg	Neg	65	0	3	Yes	85	40	1c	0	IA
OSGC2	58	NST	Pos	Neg	33	1+	2	No	40	40	2	0	IIA
OSGC3	65	Metaplastic	Neg	Neg	70	0	3	No	15	75	1c	0	IA
OSGC4	35	NST	Neg	Neg	30	1+	3	Yes	25	60	4b	2a	IIIB
OSGC5	53	NST	Neg	Neg	90	0	3	No	15	55	3	0	IIB
OSGC6	61	NST	Neg	Neg	60	0	3	No	18	35	3	0	IIB

Abbreviations: ER, estrogen receptor; PgR, progesterone receptor; LVI, lymphovascular invasion; TILs, tumor-infiltrating lymphocytes; TAMs, tumor-associated macrophages; NST, no special type.

### Recurrent miRNA signatures between OSGC and breast cancer cells

**FIGURE 2:**
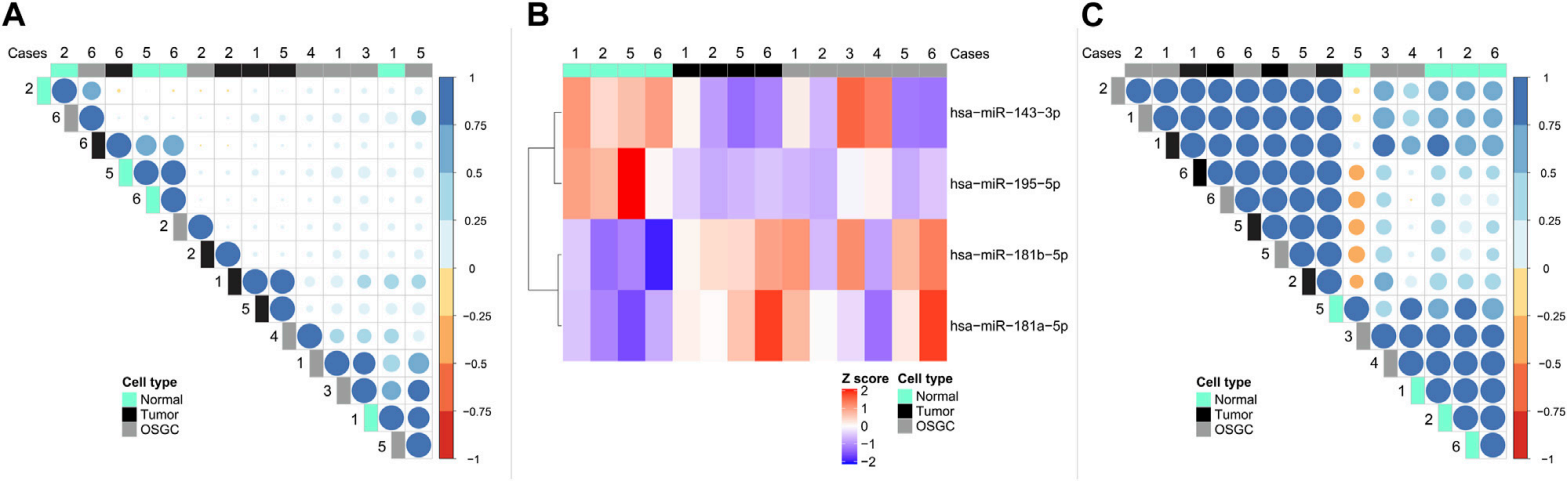
miRNA signatures in the different cellular compartments of breast cancers with OSGC. **(A)** Unsupervised separate analysis of microdissected samples of normal breast epithelium, cancer cells, and OSGC, showing that OSGC have miRNA profiles more similar to cancer samples rather than to normal cells. **(B)** Differentially expressed miRNAs between cancer cells and normal epithelial cells in breast cancers with OSGC. **(C)** OSGC grouping together with neoplastic cells tissues based on miR-181a-5p/miR-181b-5p upregulation and miR-143a-3p/miR-195a-5p downregulation in the neoplastic cells compared to normal breast epithelium.

From each of the 6 cases, the neoplastic cells, OSGC, and breast normal cells containing non-neoplastic terminal duct-lobular units, were separately microdissected, for a total number of 18 samples (i.e., 3 samples for each case). Samples failing to reach the optimal RNA quality were excluded from subsequent analyses. Altogether, a total of 6 OSGC, 4 normal, and 4 tumors microdissected samples were analyzed (*n* = 14). Applying the Pearson similarity analysis, we could document that the majority (4 out 6; 67%) of OSGC clustered together separately from cancer samples and non-neoplastic breast epithelium. By unsupervised separate analysis we asked whether the miRNA found within OSGC samples are more similar to cancer or to the non-neoplastic breast miRNAs. Considering all available miRNAs (*n* = 130) we found that OSGC had a more similar miRNA expression pattern to cancer samples, as shown by the dot color and size (Figure 2A). Next, we searched for miRNAs that were differentially expressed between cancer and non-neoplastic breast samples and we found four dysregulated miRNAs (i.e., miR-181a-5p and miR-181b-5p were upregulated, while miR-143a-3p and miR-195a-5p were significantly downregulated in the neoplastic cells compared to the non-neoplastic ones (L2R>|2|; *p* = 0.02)) (Figure 2B). Subsequently by considering these four miRNAs, we tested whether OSCG is more similar to the cancer cells or normal breast epithelial cells. According to Pearson analysis, the majority (4 out of 6; 67%) OSGCs grouped together with neoplastic breast tissue (Figure 2C). These results suggest that recurrent epigenetic events may take place in the neoplastic component affecting the OSGC molecular phenotype within the breast TIME.

### Expression of monocyte-related miRNAs in OSGCs

**FIGURE 3:**
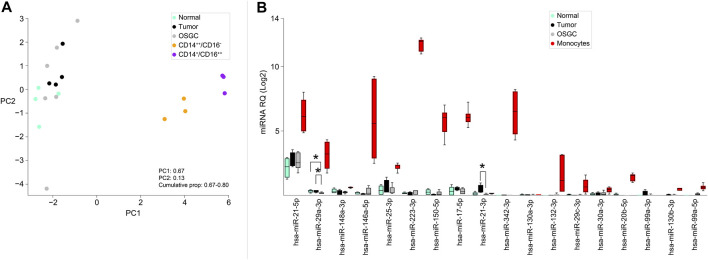
Monocyte-related miRNAs expressed in OSGCs. **(A)** Principal component analysis (PCA), unsupervised analysis of classical (CD14++/CD16−) and non-classical (CD14+/CD16++) monocyte subsets clustering separately from the normal breast epithelium, cancer cells, and OSGC. **(B)** Monocyte-specific miRNAs signature applied to breast samples in which OSGC show differential expression of miR-29a-3p and miR-21-3p.

To test the hypothesis of the monocytic origin of OSGC cells, we retrieved a publicly available miRNA signature characteristics of classical (CD14++/CD16−) and non-classical (CD14+/CD16++) monocyte subsets and we applied those signatures to our samples (Duroux-Richard et al., 2019). Generally, monocytes of either subtypes clustered separately from normal/neoplastic breast samples and OSGCs (Figure 3A). Then, the previously identified monocytes-specific miRNA signature composed of 18 miRNAs (Duroux-Richard et al., 2019) was investigated in our series (Figure 3B). The monocyte-associated miR-29a-3p and miR-21-3p were different in OSGCs compared with non-neoplastic or breast cancer tissues. Specifically, the relative expression of miR-29a-3p was lower in OSGC than in tumor and normal tissue samples, while the relative expression for miR-21-3p was lower in OSGC compared with cancer samples. Nevertheless, the remaining monocytes-associated miRNAs were not differentially modulated in breast OSGC cells. Furthermore, the signatures of monocyte-related miRNAs were completely different between the two monocytic populations and the OSGC compartment. Even though OSGC share some phenotypic similarities (i.e., CD68) with TAMs and monocytes, this cellular compartment significantly diverges from the latter when the expression of monocytic miRNA is considered.

## Discussion

Here, we analyzed the TIME composition along the miRNAome of the different cellular elements within breast cancers with OSGC. Our analyses show that these tumors are enriched in both TILs and TAMs, indicating an activation of the anti-tumor immune response. Furthermore, we provide previously unavailable evidence that OSGC are more similar to the breast neoplastic cells than to the non-neoplastic epithelial counterpart in terms of miRNA expression. This observation suggests that shared epigenetic events might occur between breast cancer tumorigenesis and OSGC phylogenesis. Finally, we demonstrated that despite OSGC show some phenotypic similarities with monocytes such as the expression of CD68 and TRAP, there is no similarity in terms of monocytic miRNA expression patterns.

The observation of OSGC within breast cancer TIME is a rare event with an unclear clinical relevance. To date, only 200 cases have been described in the literature; for this reason, their biology is substantially undetermined (Zhou et al., 2014). The World Health Organization (WHO) classifies the carcinoma with OSGC among rare variants of invasive breast carcinomas NST (WHO Classification of Tumours Editorial Board and WHO Classification of Breast Tumours, 2019). The carcinomatous part of these lesions is most frequently described as a well-to-moderately differentiated (Zhou et al., 2014; Ohashi et al., 2018a). In our study group, however, the majority (*n* = 5, 83.3%) of cases were mostly poorly differentiated invasive breast carcinomas NST, although metaplastic features were seen in one case. Our observation confirms the great morphological heterogeneity of these neoplasms, where a wide range of breast cancer special types [e.g., cribriform, mucinous (micro) papillary, lobular, and metaplastic] with variable histological grades can be accompanied by OSGC (Marchiò et al., 2015; Ohashi et al., 2018a; Bonsang et al., 2019; Hoda et al., 2020). Previous studies on solid tumors associated with OSGC have unraveled that these cells have a histiocytic origin based on their phenotype and molecular features (Bauditz et al., 2006; Dahm, 2017). To the best of our knowledge, there is an extremely limited information regarding the specific composition of the TIME in breast cancer with OSGC, thus, the information provided in our study could constitute the basis for additional research focused on the correlation of this morphological features with the clinical course, tumor aggressiveness, and application of therapeutic strategies (Pruneri et al., 2016; Sajjadi et al., 2020; Criscitiello et al., 2021). By analyzing the composition of TIME, we observed the steady presence of TILs and high TAMs within the TIME of all cases, providing circumstantial evidence to suggest that OSGC might be a contributor to the host anti-tumor immunity. In addition, we demonstrated for the first time in the literature that the OSGC miRNA expression landscape shares similar characteristics with that of breast cancer cells. Hence, in our microdissected samples, we found that OSGC could be grouped with cancer cells based on four markers, namely hsa-miR-181a-5p, hsa-miR-181b-5p, hsa-miR-143-3p, and hsa-miR-195-5p. These miRNAs have been previously described as regulators of genes involved in breast cancer tumorigenesis and immune infiltration (Allantaz et al., 2012; Alsaweed et al., 2015; Johannessen et al., 2017; Liu et al., 2017; Rizzo et al., 2017; Li et al., 2021). Among these miRNAs, interestingly, miR-143 regulates the machinery of the breast epigenome (Ng et al., 2014; Humphries et al., 2019). In breast cancer, miR-143 directly targets and regulates DNMT3A. DNMT3A overexpression could alter the methylation status of PTEN and TNFRSF10C which contribute to tumorigenesis (Ng et al., 2014; Humphries et al., 2019). This highlights the tumor-suppressive role of miR-143 in epigenetic aberration of breast cancer.

To assess whether OSGCs are biologically related to TAMs, we performed targeted miRNA expression profiling of monocyte signatures in the different cellular components of our samples. Altogether, we found two markers that were differentially expressed within the OSGC, namely miR-29a-3p and miR-21-3p. Interestingly, miR29a-3p has been recently reported to be involved in the regulation of TAMs-derived exosomal long non-coding (lnc) RNAs that can promote proliferation, invasion, and restrain cell apoptosis in several conditions associated with abnormal osteoclast-mediated osteolysis, such as osteosarcoma, osteoporosis, bone loss, and breast cancer metastatic to the bone (Lian et al., 2019; Wu et al., 2019; de Sire et al., 2020; Cascini and Chiodoni, 2021; Hrdlicka et al., 2021; Nørregaard et al., 2021; Venetis et al., 2021; Zhang et al., 2021). In addition, several studies have documented that miR-21 is the most abundant miRNA in macrophages and TAMs (Wang et al., 2015a; Canfrán-Duque et al., 2017). In contrast to macrophage function in normal tissue, TAMs are classified into two major distinctive phenotypes: the pro-inflammatory (tumoricidal) M1 macrophages and immunosuppressive (tumor-promoting) M2 macrophages (Rivera and Bergers, 2013). In particular, miR-21 is considered as a homeostatic regulator of macrophage differentiation, as its deficiency prompts M1 polarization in TAMs (Xi et al., 2018). For this reason, tumor cells may stimulate miR-21 expression in TAMs to prevent tumoricidal M1 polarization (Sahraei et al., 2019). Recently, it has been observed that miR-21 increases the M2 macrophage-mediated chemoresistance and downregulates major histocompatibility complex (MHC) class I surface antigens, while upregulating programmed death-ligand 1 (PD-L1) expression in TAMs, which is known to inhibit phagocytic anti-tumor activity (Caescu et al., 2015; An and Yang, 2020; Subbarayan et al., 2021). This negative regulator of inflammation and phosphatase and tensin homolog (PTEN)/phosphoinositide 3-kinase (PI3K) axis has also been studied for its role in balancing apoptosis and oncogenic transformation in normal epithelial cells and as a prognostic biomarker in breast cancer (Buscaglia and Li, 2011; Wang et al., 2015b; Ma et al., 2015; Fusco et al., 2020b; Amirfallah et al., 2021; Fusco et al., 2021; Sajjadi et al., 2021). Interestingly, we found several similarities between OSGC and M2-TAMs, particularly in their morphology and immunophenotype, and a miRNA monocytic signature.

Our study has several limitations. First, given the rarity of breast cancers with OSGC, we could only analyze a relatively small number of cases, thus this study should be regarded as hypothesis-generating. It should be noted, however, that this study, to the best of our knowledge, represents the first integrated miRNA analysis of breast cancer and associated OSGC. Second, given the relatively limited quantity of OSGC and that all cases were microdissected from sections obtained from formalin-fixed paraffin-embedded (FFPE) blocks, we could only perform targeted miRNA expression profiling; hence, potential somatic genetic alterations and dysregulations affecting miRNAs not included in the panel studied could play a role in the development of OSGC and modulation of TILs and TAMs within breast cancer TIME. Finally, due to the retrospective nature of this cohort, risk and survival analyses have not been performed. Large prospective multicentric studies are needed to investigate the specific outcome of breast cancer with OSGC and the risk of development of systemic (including bone) metastases conferred by the presence of OSGC. In this respect, it would be of great interest to perform multi-level high-throughput analyses, coupled with bioinformatics investigations, to include selected miRNA and predicted target genes accompanied by functional analysis and GO terms clustering, in order to assess further differences and similarities among immune and cancer cells, in different tumor types.

Despite these limitations, this study, together with previous observations, challenges the notion that OSGC is a mere histologic curiosity within the wide spectrum of breast cancer. Larger multicentric clinical studies and cancer registries, coupled with comprehensive molecular analyses will be required to 1) determine whether OSGC may constitute a prognostic/predictive biomarker, 2) identify potential founder genetic/epigenetic events in these tumors, and 3) to define whether breast cancers with OSGC would be associated with a hyperactivated anti-tumor immune response.

In conclusion, our findings on the presence of stromal TILs with a high proportion of TAMs suggest an activated TIME in breast cancer with OSGC. We found that OSGC miRNAs profile were more similar to cancer cells than to non-neoplastic epithelial counterparts implying the potential role of epigenetic events on both neoplastic and OSGC component. Finally, all OSGC within each case displayed CD68 expression and partially a monocytes’ miRNA signature, suggesting that OSGC might be resident elements of TIME and likely belong to the spectrum of immunosuppressive, tumor-promoting, M2 TAMs.

## Data Availability

The raw data supporting the conclusions of this article will be made available by the authors, without undue reservation, upon reasonable request.
