# Supplementary material for: Osteoclast-like stromal giant cells in breast cancer likely belong to the spectrum of immunosuppressive tumor-associated macrophages
Source: Front Mol Biosci. 2022 Aug 26;9:894247. doi: 10.3389/fmolb.2022.894247 (PMC9462457; doi:10.3389/fmolb.2022.894247)
Supplement: Supplementary file 1 [file Table1.DOCX]

| **Supplementary Table S1.** List of the miRNAs analyzed. | | | | | | | |
| --- | --- | --- | --- | --- | --- | --- | --- |
|  |  |  |  |  |  |  |  |
| 1 | hsa-let-7a-3p-477861_mir | 51 | hsa-miR-1225-3p-477875_mir | 101 | hsa-miR-1285-3p-478687_mir | 151 | hsa-miR-145-5p-477916_mir |
| 2 | hsa-let-7a-5p-478575_mir | 52 | hsa-miR-1226-5p-478641_mir | 102 | hsa-miR-1286-477893_mir | 152 | hsa-miR-146a-3p-478714_mir |
| 3 | hsa-let-7b-3p-478221_mir | 53 | hsa-miR-1227-3p-478642_mir | 103 | hsa-miR-1288-3p-478688_mir | 153 | hsa-miR-146a-5p-478399_mir |
| 4 | hsa-let-7b-5p-478576_mir | 54 | hsa-miR-1228-5p-478644_mir | 104 | hsa-miR-1289-478689_mir | 154 | hsa-miR-146b-3p-478715_mir |
| 5 | hsa-let-7c-5p-478577_mir | 55 | hsa-miR-1233-3p-477877_mir | 105 | hsa-miR-129-1-3p-480873_mir | 155 | hsa-miR-146b-5p-478513_mir |
| 6 | hsa-let-7d-3p-477848_mir | 56 | hsa-miR-1236-3p-478647_mir | 106 | hsa-miR-129-2-3p-478544_mir | 156 | hsa-miR-147a-478514_mir |
| 7 | hsa-let-7d-5p-478439_mir | 57 | hsa-miR-1238-3p-478649_mir | 107 | hsa-miR-129-5p-477896_mir | 157 | hsa-miR-147b-478717_mir |
| 8 | hsa-let-7e-3p-479281_mir | 58 | hsa-miR-124-3p-477879_mir | 108 | hsa-miR-1290-477895_mir | 158 | hsa-miR-148a-3p-477814_mir |
| 9 | hsa-let-7e-5p-478579_mir | 59 | hsa-miR-124-5p-477880_mir | 109 | hsa-miR-1291-478690_mir | 159 | hsa-miR-148a-5p-478718_mir |
| 10 | hsa-let-7f-1-3p-477801_mir | 60 | hsa-miR-1243-478650_mir | 110 | hsa-miR-1292-5p-478691_mir | 160 | hsa-miR-148b-3p-477824_mir |
| 11 | hsa-let-7f-2-3p-477843_mir | 61 | hsa-miR-1244-478651_mir | 111 | hsa-miR-1293-478692_mir | 161 | hsa-miR-148b-5p-478719_mir |
| 12 | hsa-let-7f-5p-478578_mir | 62 | hsa-miR-1245a-478652_mir | 112 | hsa-miR-1294-478693_mir | 162 | hsa-miR-149-3p-478720_mir |
| 13 | hsa-let-7g-3p-477850_mir | 63 | hsa-miR-1247-5p-477882_mir | 113 | hsa-miR-1296-5p-479451_mir | 163 | hsa-miR-149-5p-477917_mir |
| 14 | hsa-let-7g-5p-478580_mir | 64 | hsa-miR-1248-478653_mir | 114 | hsa-miR-1298-5p-479452_mir | 164 | hsa-miR-150-3p-478721_mir |
| 15 | hsa-let-7i-3p-477862_mir | 65 | hsa-miR-1249-3p-478654_mir | 115 | hsa-miR-1301-3p-477897_mir | 165 | hsa-miR-150-5p-477918_mir |
| 16 | hsa-let-7i-5p-478375_mir | 66 | hsa-miR-1250-5p-478655_mir | 116 | hsa-miR-1302-478697_mir | 166 | hsa-miR-151a-3p-477919_mir |
| 17 | hsa-miR-1-3p-477820_mir | 67 | hsa-miR-1251-5p-478657_mir | 117 | hsa-miR-1303-478698_mir | 167 | hsa-miR-151a-5p-478505_mir |
| 18 | hsa-miR-100-3p-478619_mir | 68 | hsa-miR-1252-5p-478658_mir | 118 | hsa-miR-1304-5p-478699_mir | 168 | hsa-miR-151b-477811_mir |
| 19 | hsa-miR-100-5p-478224_mir | 69 | hsa-miR-1253-478659_mir | 119 | hsa-miR-130a-3p-477851_mir | 169 | hsa-miR-152-3p-477921_mir |
| 20 | hsa-miR-101-3p-477863_mir | 70 | hsa-miR-1254-478660_mir | 120 | hsa-miR-130a-5p-478702_mir | 170 | hsa-miR-153-3p-477922_mir |
| 21 | hsa-miR-101-5p-478620_mir | 71 | hsa-miR-1255a-478661_mir | 121 | hsa-miR-130b-3p-477840_mir | 171 | hsa-miR-154-3p-478725_mir |
| 22 | hsa-miR-103a-2-5p-477864_mir | 72 | hsa-miR-1255b-5p-478662_mir | 122 | hsa-miR-130b-5p-477899_mir | 172 | hsa-miR-154-5p-477925_mir |
| 23 | hsa-miR-103a-3p-478253_mir | 73 | hsa-miR-1256-478663_mir | 123 | hsa-miR-132-3p-477900_mir | 173 | hsa-miR-155-3p-477926_mir |
| 24 | hsa-miR-105-3p-478622_mir | 74 | hsa-miR-1257-478664_mir | 124 | hsa-miR-132-5p-478705_mir | 174 | hsa-miR-155-5p-477927_mir |
| 25 | hsa-miR-105-5p-477865_mir | 75 | hsa-miR-125a-3p-477883_mir | 125 | hsa-miR-1324-478704_mir | 175 | hsa-miR-15a-3p-477928_mir |
| 26 | hsa-miR-106a-3p-478623_mir | 76 | hsa-miR-125a-5p-477884_mir | 126 | hsa-miR-133a-3p-478511_mir | 176 | hsa-miR-15a-5p-477858_mir |
| 27 | hsa-miR-106a-5p-478225_mir | 77 | hsa-miR-125b-1-3p-478665_mir | 127 | hsa-miR-133b-480871_mir | 177 | hsa-miR-15b-3p-477929_mir |
| 28 | hsa-miR-106b-3p-477866_mir | 78 | hsa-miR-125b-2-3p-478666_mir | 128 | hsa-miR-134-5p-477901_mir | 178 | hsa-miR-15b-5p-478313_mir |
| 29 | hsa-miR-106b-5p-478412_mir | 79 | hsa-miR-125b-5p-477885_mir | 129 | hsa-miR-135a-5p-478581_mir | 179 | hsa-miR-16-1-3p-478727_mir |
| 30 | hsa-miR-107-478254_mir | 80 | hsa-miR-126-3p-477887_mir | 130 | hsa-miR-135b-3p-478710_mir | 180 | hsa-miR-16-2-3p-477931_mir |
| 31 | hsa-miR-10a-3p-478624_mir | 81 | hsa-miR-126-5p-477888_mir | 131 | hsa-miR-135b-5p-478582_mir | 181 | hsa-miR-16-5p-477860_mir |
| 32 | hsa-miR-10a-5p-479241_mir | 82 | hsa-miR-1260a-478476_mir | 132 | hsa-miR-136-3p-477902_mir | 182 | hsa-miR-17-3p-477932_mir |
| 33 | hsa-miR-10b-3p-477868_mir | 83 | hsa-miR-1262-478668_mir | 133 | hsa-miR-136-5p-478307_mir | 183 | hsa-miR-17-5p-478447_mir |
| 34 | hsa-miR-10b-5p-478494_mir | 84 | hsa-miR-1263-478669_mir | 134 | hsa-miR-137-477904_mir | 184 | hsa-miR-181a-2-3p-479478_mir |
| 35 | hsa-miR-1178-3p-478625_mir | 85 | hsa-miR-1264-478670_mir | 135 | hsa-miR-138-1-3p-478311_mir | 185 | hsa-miR-181a-3p-479405_mir |
| 36 | hsa-miR-1179-478626_mir | 86 | hsa-miR-1265-478671_mir | 136 | hsa-miR-138-2-3p-478711_mir | 186 | hsa-miR-181a-5p-477857_mir |
| 37 | hsa-miR-1180-3p-477869_mir | 87 | hsa-miR-1267-478672_mir | 137 | hsa-miR-138-5p-477905_mir | 187 | hsa-miR-181b-5p-478583_mir |
| 38 | hsa-miR-1182-478628_mir | 88 | hsa-miR-1269a-479522_mir | 138 | hsa-miR-139-3p-477906_mir | 188 | hsa-miR-181c-3p-477933_mir |
| 39 | hsa-miR-1183-477870_mir | 89 | hsa-miR-127-3p-477889_mir | 139 | hsa-miR-139-5p-478312_mir | 189 | hsa-miR-181c-5p-477934_mir |
| 40 | hsa-miR-1184-478629_mir | 90 | hsa-miR-127-5p-477891_mir | 140 | hsa-miR-140-3p-477908_mir | 190 | hsa-miR-181d-5p-479517_mir |
| 41 | hsa-miR-1197-478630_mir | 91 | hsa-miR-1270-478673_mir | 141 | hsa-miR-140-5p-477909_mir | 191 | hsa-miR-182-3p-478729_mir |
| 42 | hsa-miR-1200-478631_mir | 92 | hsa-miR-1271-5p-478674_mir | 142 | hsa-miR-141-3p-478501_mir | 192 | hsa-miR-182-5p-477935_mir |
| 43 | hsa-miR-1203-478310_mir | 93 | hsa-miR-1272-478675_mir | 143 | hsa-miR-141-5p-478712_mir | 193 | hsa-miR-1825-478730_mir |
| 44 | hsa-miR-1204-478633_mir | 94 | hsa-miR-1275-477890_mir | 144 | hsa-miR-142-3p-477910_mir | 194 | hsa-miR-183-3p-477936_mir |
| 45 | hsa-miR-1205-478634_mir | 95 | hsa-miR-1276-478680_mir | 145 | hsa-miR-142-5p-477911_mir | 195 | hsa-miR-183-5p-477937_mir |
| 46 | hsa-miR-1206-478635_mir | 96 | hsa-miR-1278-478681_mir | 146 | hsa-miR-143-3p-477912_mir | 196 | hsa-miR-184-477938_mir |
| 47 | hsa-miR-1208-478637_mir | 97 | hsa-miR-128-3p-477892_mir | 147 | hsa-miR-143-5p-478713_mir | 197 | hsa-miR-185-3p-478732_mir |
| 48 | hsa-miR-122-3p-477874_mir | 98 | hsa-miR-1282-478683_mir | 148 | hsa-miR-144-3p-477913_mir | 198 | hsa-miR-185-5p-477939_mir |
| 49 | hsa-miR-122-5p-477855_mir | 99 | hsa-miR-1283-478685_mir | 149 | hsa-miR-144-5p-477914_mir | 199 | hsa-miR-186-3p-479544_mir |
| 50 | hsa-miR-1224-3p-478638_mir | 100 | hsa-miR-1284-478686_mir | 150 | hsa-miR-145-3p-477915_mir | 200 | hsa-miR-186-5p-477940_mir |

| 201 | hsa-miR-187-3p-477941_mir | 251 | hsa-miR-20a-5p-478586_mir | 301 | hsa-miR-296-3p-478790_mir | 351 | hsa-miR-331-5p-478032_mir |
| --- | --- | --- | --- | --- | --- | --- | --- |
| 202 | hsa-miR-188-3p-477942_mir | 252 | hsa-miR-20b-3p-478764_mir | 302 | hsa-miR-296-5p-477836_mir | 352 | hsa-miR-335-3p-478033_mir |
| 203 | hsa-miR-188-5p-477943_mir | 253 | hsa-miR-20b-5p-477804_mir | 303 | hsa-miR-298-478430_mir | 353 | hsa-miR-335-5p-478324_mir |
| 204 | hsa-miR-18a-3p-477944_mir | 254 | hsa-miR-21-3p-477973_mir | 304 | hsa-miR-299-3p-478792_mir | 354 | hsa-miR-337-3p-478035_mir |
| 205 | hsa-miR-18a-5p-478551_mir | 255 | hsa-miR-21-5p-477975_mir | 305 | hsa-miR-299-5p-478793_mir | 355 | hsa-miR-337-5p-478036_mir |
| 206 | hsa-miR-18b-3p-478734_mir | 256 | hsa-miR-210-3p-477970_mir | 306 | hsa-miR-29a-3p-478587_mir | 356 | hsa-miR-338-3p-478037_mir |
| 207 | hsa-miR-18b-5p-478584_mir | 257 | hsa-miR-211-5p-478507_mir | 307 | hsa-miR-29a-5p-478002_mir | 357 | hsa-miR-338-5p-478038_mir |
| 208 | hsa-miR-190a-5p-478358_mir | 258 | hsa-miR-2110-477971_mir | 308 | hsa-miR-29b-1-5p-478794_mir | 358 | hsa-miR-339-3p-478325_mir |
| 209 | hsa-miR-190b-477948_mir | 259 | hsa-miR-212-3p-478318_mir | 309 | hsa-miR-29b-2-5p-478003_mir | 359 | hsa-miR-339-5p-478040_mir |
| 210 | hsa-miR-191-3p-477951_mir | 260 | hsa-miR-214-3p-477974_mir | 310 | hsa-miR-29b-3p-478369_mir | 360 | hsa-miR-33a-3p-478831_mir |
| 211 | hsa-miR-191-5p-477952_mir | 261 | hsa-miR-214-5p-478768_mir | 311 | hsa-miR-29c-3p-479229_mir | 361 | hsa-miR-33a-5p-478347_mir |
| 212 | hsa-miR-192-3p-478741_mir | 262 | hsa-miR-215-5p-478516_mir | 312 | hsa-miR-29c-5p-478005_mir | 362 | hsa-miR-33b-5p-478479_mir |
| 213 | hsa-miR-192-5p-478262_mir | 263 | hsa-miR-216a-5p-477976_mir | 313 | hsa-miR-301a-3p-477815_mir | 363 | hsa-miR-340-3p-478041_mir |
| 214 | hsa-miR-193a-3p-478306_mir | 264 | hsa-miR-216b-5p-479456_mir | 314 | hsa-miR-301b-3p-477825_mir | 364 | hsa-miR-340-5p-478042_mir |
| 215 | hsa-miR-193a-5p-477954_mir | 265 | hsa-miR-217-478773_mir | 315 | hsa-miR-302a-3p-478006_mir | 365 | hsa-miR-342-3p-478043_mir |
| 216 | hsa-miR-193b-3p-478314_mir | 266 | hsa-miR-218-1-3p-478774_mir | 316 | hsa-miR-302a-5p-478798_mir | 366 | hsa-miR-342-5p-478044_mir |
| 217 | hsa-miR-193b-5p-478742_mir | 267 | hsa-miR-218-2-3p-478775_mir | 317 | hsa-miR-302b-3p-478591_mir | 367 | hsa-miR-345-5p-478366_mir |
| 218 | hsa-miR-194-3p-478743_mir | 268 | hsa-miR-218-5p-477977_mir | 318 | hsa-miR-302b-5p-478799_mir | 368 | hsa-miR-346-478046_mir |
| 219 | hsa-miR-194-5p-477956_mir | 269 | hsa-miR-219a-1-3p-477978_mir | 319 | hsa-miR-302c-3p-478509_mir | 369 | hsa-miR-34a-3p-478047_mir |
| 220 | hsa-miR-195-3p-478744_mir | 270 | hsa-miR-219a-2-3p-477979_mir | 320 | hsa-miR-302c-5p-478800_mir | 370 | hsa-miR-34a-5p-478048_mir |
| 221 | hsa-miR-195-5p-477957_mir | 271 | hsa-miR-219a-5p-477980_mir | 321 | hsa-miR-302d-3p-478237_mir | 371 | hsa-miR-34b-3p-478049_mir |
| 222 | hsa-miR-196a-3p-478745_mir | 272 | hsa-miR-22-3p-477985_mir | 322 | hsa-miR-302d-5p-478801_mir | 372 | hsa-miR-34b-5p-478050_mir |
| 223 | hsa-miR-196a-5p-478230_mir | 273 | hsa-miR-22-5p-477987_mir | 323 | hsa-miR-30a-3p-478273_mir | 373 | hsa-miR-34c-3p-478051_mir |
| 224 | hsa-miR-196b-3p-477958_mir | 274 | hsa-miR-221-3p-477981_mir | 324 | hsa-miR-30a-5p-479448_mir | 374 | hsa-miR-34c-5p-478052_mir |
| 225 | hsa-miR-196b-5p-478585_mir | 275 | hsa-miR-221-5p-478778_mir | 325 | hsa-miR-30b-3p-478804_mir | 375 | hsa-miR-361-3p-478055_mir |
| 226 | hsa-miR-197-3p-477959_mir | 276 | hsa-miR-222-3p-477982_mir | 326 | hsa-miR-30b-5p-478007_mir | 376 | hsa-miR-361-5p-478056_mir |
| 227 | hsa-miR-198-478749_mir | 277 | hsa-miR-222-5p-478779_mir | 327 | hsa-miR-30c-1-3p-479412_mir | 377 | hsa-miR-362-3p-478058_mir |
| 228 | hsa-miR-199a-3p_hsa-miR-199b-3p-477961_mir | 278 | hsa-miR-223-3p-477983_mir | 328 | hsa-miR-30c-2-3p-479401_mir | 378 | hsa-miR-362-5p-478059_mir |
| 229 | hsa-miR-199a-5p-478231_mir | 279 | hsa-miR-223-5p-477984_mir | 329 | hsa-miR-30c-5p-478008_mir | 379 | hsa-miR-363-3p-478060_mir |
| 230 | hsa-miR-199b-5p-478486_mir | 280 | hsa-miR-224-5p-477986_mir | 330 | hsa-miR-30d-3p-479362_mir | 380 | hsa-miR-363-5p-478840_mir |
| 231 | hsa-miR-19a-3p-479228_mir | 281 | hsa-miR-23a-3p-478532_mir | 331 | hsa-miR-30d-5p-478606_mir | 381 | hsa-miR-365a-3p_hsa-miR-365b-3p-478065_mir |
| 232 | hsa-miR-19a-5p-478750_mir | 282 | hsa-miR-23a-5p-478782_mir | 332 | hsa-miR-30e-3p-478388_mir | 382 | hsa-miR-367-3p-478066_mir |
| 233 | hsa-miR-19b-1-5p-477962_mir | 283 | hsa-miR-23b-3p-478602_mir | 333 | hsa-miR-30e-5p-479235_mir | 383 | hsa-miR-367-5p-478846_mir |
| 234 | hsa-miR-19b-3p-478264_mir | 284 | hsa-miR-23b-5p-477991_mir | 334 | hsa-miR-31-3p-478012_mir | 384 | hsa-miR-369-3p-478067_mir |
| 235 | hsa-miR-200a-3p-478490_mir | 285 | hsa-miR-24-1-5p-478784_mir | 335 | hsa-miR-31-5p-478015_mir | 385 | hsa-miR-369-5p-478068_mir |
| 236 | hsa-miR-200a-5p-478752_mir | 286 | hsa-miR-24-2-5p-478785_mir | 336 | hsa-miR-32-3p-478827_mir | 386 | hsa-miR-370-3p-478326_mir |
| 237 | hsa-miR-200b-3p-477963_mir | 287 | hsa-miR-24-3p-477992_mir | 337 | hsa-miR-32-5p-478026_mir | 387 | hsa-miR-371a-3p-478070_mir |
| 238 | hsa-miR-200b-5p-478753_mir | 288 | hsa-miR-25-3p-477994_mir | 338 | hsa-miR-320a-478594_mir | 388 | hsa-miR-372-3p-478071_mir |
| 239 | hsa-miR-200c-3p-478351_mir | 289 | hsa-miR-25-5p-478786_mir | 339 | hsa-miR-320b-478588_mir | 389 | hsa-miR-373-3p-478363_mir |
| 240 | hsa-miR-200c-5p-478754_mir | 290 | hsa-miR-26a-1-3p-478787_mir | 340 | hsa-miR-323a-3p-477853_mir | 390 | hsa-miR-374a-3p-478855_mir |
| 241 | hsa-miR-202-3p-478417_mir | 291 | hsa-miR-26a-2-3p-478788_mir | 341 | hsa-miR-323b-5p-478826_mir | 391 | hsa-miR-374a-5p-478238_mir |
| 242 | hsa-miR-202-5p-478755_mir | 292 | hsa-miR-26a-5p-477995_mir | 342 | hsa-miR-324-3p-478023_mir | 392 | hsa-miR-374b-3p-479421_mir |
| 243 | hsa-miR-203a-3p-478316_mir | 293 | hsa-miR-26b-3p-477996_mir | 343 | hsa-miR-324-5p-478024_mir | 393 | hsa-miR-374b-5p-478389_mir |
| 244 | hsa-miR-203b-5p-478758_mir | 294 | hsa-miR-26b-5p-478418_mir | 344 | hsa-miR-325-478025_mir | 394 | hsa-miR-375-478074_mir |
| 245 | hsa-miR-204-5p-478491_mir | 295 | hsa-miR-27a-3p-478384_mir | 345 | hsa-miR-326-478027_mir | 395 | hsa-miR-376a-3p-478240_mir |
| 246 | hsa-miR-205-5p-477967_mir | 296 | hsa-miR-27a-5p-477998_mir | 346 | hsa-miR-328-3p-478028_mir | 396 | hsa-miR-376a-5p-478859_mir |
| 247 | hsa-miR-206-477968_mir | 297 | hsa-miR-27b-3p-478270_mir | 347 | hsa-miR-329-3p-478029_mir | 397 | hsa-miR-376b-3p-478860_mir |
| 248 | hsa-miR-208a-3p-477819_mir | 298 | hsa-miR-27b-5p-478789_mir | 348 | hsa-miR-330-3p-478030_mir | 398 | hsa-miR-376c-3p-478459_mir |
| 249 | hsa-miR-208b-3p-477806_mir | 299 | hsa-miR-28-3p-477999_mir | 349 | hsa-miR-330-5p-478830_mir | 399 | hsa-miR-377-3p-478075_mir |
| 250 | hsa-miR-20a-3p-478317_mir | 300 | hsa-miR-28-5p-478000_mir | 350 | hsa-miR-331-3p-478323_mir | 400 | hsa-miR-377-5p-478863_mir |

| 401 | hsa-miR-378a-3p-478349_mir | 451 | hsa-miR-487b-3p-477835_mir | 501 | hsa-miR-518c-3p-478982_mir | 551 | hsa-miR-548d-5p-480870_mir |
| --- | --- | --- | --- | --- | --- | --- | --- |
| 402 | hsa-miR-378a-5p-478076_mir | 452 | hsa-miR-488-3p-478129_mir | 502 | hsa-miR-518c-5p-478983_mir | 552 | hsa-miR-548e-3p-478362_mir |
| 403 | hsa-miR-379-5p-478077_mir | 453 | hsa-miR-488-5p-478939_mir | 503 | hsa-miR-518d-3p-479393_mir | 553 | hsa-miR-548g-3p-479020_mir |
| 404 | hsa-miR-380-3p-477854_mir | 454 | hsa-miR-489-3p-478130_mir | 504 | hsa-miR-518d-5p_hsa-miR-520c-5p_hsa-miR-526a-479530_mir | 554 | hsa-miR-548h-5p-479503_mir |
| 405 | hsa-miR-380-5p-478865_mir | 455 | hsa-miR-490-3p-478131_mir | 505 | hsa-miR-518e-3p-479408_mir | 555 | hsa-miR-548i-480874_mir |
| 406 | hsa-miR-381-3p-477816_mir | 456 | hsa-miR-491-3p-478942_mir | 506 | hsa-miR-518e-5p_hsa-miR-519a-5p_ | 556 | hsa-miR-548j-5p-479022_mir |
| 407 | hsa-miR-382-5p-478078_mir | 457 | hsa-miR-491-5p-478132_mir | 507 | hsa-miR-518f-3p-478984_mir | 557 | hsa-miR-548k-479374_mir |
| 408 | hsa-miR-383-5p-478079_mir | 458 | hsa-miR-492-478133_mir | 508 | hsa-miR-518f-5p-479532_mir | 558 | hsa-miR-548l-479391_mir |
| 409 | hsa-miR-384-478080_mir | 459 | hsa-miR-493-3p-478134_mir | 509 | hsa-miR-519a-3p-479534_mir | 559 | hsa-miR-548m-479023_mir |
| 410 | hsa-miR-409-3p-478084_mir | 460 | hsa-miR-494-3p-478135_mir | 510 | hsa-miR-519b-3p-479333_mir | 560 | hsa-miR-548n-479024_mir |
| 411 | hsa-miR-409-5p-478872_mir | 461 | hsa-miR-495-3p-478136_mir | 511 | hsa-miR-519c-3p-479495_mir | 561 | hsa-miR-548p-479025_mir |
| 412 | hsa-miR-410-3p-478085_mir | 462 | hsa-miR-496-478335_mir | 512 | hsa-miR-519d-3p-478986_mir | 562 | hsa-miR-549a-479030_mir |
| 413 | hsa-miR-411-3p-479526_mir | 463 | hsa-miR-497-3p-478946_mir | 513 | hsa-miR-519e-3p-479340_mir | 563 | hsa-miR-550a-3p-479032_mir |
| 414 | hsa-miR-411-5p-478086_mir | 464 | hsa-miR-497-5p-478138_mir | 514 | hsa-miR-519e-5p-478988_mir | 564 | hsa-miR-550a-5p-477852_mir |
| 415 | hsa-miR-412-3p-478087_mir | 465 | hsa-miR-499a-3p-478948_mir | 515 | hsa-miR-520a-3p-478989_mir | 565 | hsa-miR-551a-478158_mir |
| 416 | hsa-miR-421-478088_mir | 466 | hsa-miR-499a-5p-478139_mir | 516 | hsa-miR-520a-5p-479404_mir | 566 | hsa-miR-551b-3p-478159_mir |
| 417 | hsa-miR-422a-478481_mir | 467 | hsa-miR-500a-3p-478951_mir | 517 | hsa-miR-520b-479509_mir | 567 | hsa-miR-551b-5p-479035_mir |
| 418 | hsa-miR-423-3p-478327_mir | 468 | hsa-miR-500a-5p-478309_mir | 518 | hsa-miR-520c-3p-479536_mir | 568 | hsa-miR-552-3p-479036_mir |
| 419 | hsa-miR-423-5p-478090_mir | 469 | hsa-miR-501-3p-478350_mir | 519 | hsa-miR-520d-3p-478990_mir | 569 | hsa-miR-553-479038_mir |
| 420 | hsa-miR-424-3p-478091_mir | 470 | hsa-miR-501-5p-478142_mir | 520 | hsa-miR-520d-5p-478616_mir | 570 | hsa-miR-554-479039_mir |
| 421 | hsa-miR-424-5p-478092_mir | 471 | hsa-miR-502-3p-478348_mir | 521 | hsa-miR-520e-478498_mir | 571 | hsa-miR-555-479040_mir |
| 422 | hsa-miR-425-3p-478093_mir | 472 | hsa-miR-502-5p-478954_mir | 522 | hsa-miR-520f-3p-479343_mir | 572 | hsa-miR-556-3p-479041_mir |
| 423 | hsa-miR-425-5p-478094_mir | 473 | hsa-miR-503-5p-478143_mir | 523 | hsa-miR-520g-3p-478991_mir | 573 | hsa-miR-556-5p-479042_mir |
| 424 | hsa-miR-429-477849_mir | 474 | hsa-miR-504-5p-478144_mir | 524 | hsa-miR-520h-479499_mir | 574 | hsa-miR-557-479043_mir |
| 425 | hsa-miR-431-3p-478888_mir | 475 | hsa-miR-505-3p-478145_mir | 525 | hsa-miR-521-478149_mir | 575 | hsa-miR-558-479044_mir |
| 426 | hsa-miR-431-5p-478889_mir | 476 | hsa-miR-505-5p-478957_mir | 526 | hsa-miR-522-3p-478993_mir | 576 | hsa-miR-559-479045_mir |
| 427 | hsa-miR-432-3p-478892_mir | 477 | hsa-miR-506-3p-478958_mir | 527 | hsa-miR-523-3p-478994_mir | 577 | hsa-miR-561-3p-478160_mir |
| 428 | hsa-miR-432-5p-478101_mir | 478 | hsa-miR-507-478960_mir | 528 | hsa-miR-524-3p-479338_mir | 578 | hsa-miR-562-479047_mir |
| 429 | hsa-miR-433-3p-478102_mir | 479 | hsa-miR-508-3p-478961_mir | 529 | hsa-miR-524-5p-479285_mir | 579 | hsa-miR-563-479048_mir |
| 430 | hsa-miR-448-478105_mir | 480 | hsa-miR-508-5p-478962_mir | 530 | hsa-miR-525-3p-478995_mir | 580 | hsa-miR-564-478161_mir |
| 431 | hsa-miR-449a-478561_mir | 481 | hsa-miR-509-3-5p-478963_mir | 531 | hsa-miR-525-5p-479396_mir | 581 | hsa-miR-566-479373_mir |
| 432 | hsa-miR-449b-5p-479528_mir | 482 | hsa-miR-509-5p-478965_mir | 532 | hsa-miR-526b-5p-478997_mir | 582 | hsa-miR-567-479049_mir |
| 433 | hsa-miR-450a-5p-478106_mir | 483 | hsa-miR-510-5p-478968_mir | 533 | hsa-miR-532-3p-478336_mir | 583 | hsa-miR-569-479051_mir |
| 434 | hsa-miR-450b-3p-478913_mir | 484 | hsa-miR-511-5p-478970_mir | 534 | hsa-miR-532-5p-478151_mir | 584 | hsa-miR-570-3p-479053_mir |
| 435 | hsa-miR-450b-5p-478914_mir | 485 | hsa-miR-512-3p-478971_mir | 535 | hsa-miR-539-5p-478152_mir | 585 | hsa-miR-571-479054_mir |
| 436 | hsa-miR-451a-478107_mir | 486 | hsa-miR-512-5p-478972_mir | 536 | hsa-miR-541-3p-478999_mir | 586 | hsa-miR-572-478162_mir |
| 437 | hsa-miR-452-3p-478917_mir | 487 | hsa-miR-513a-5p-479483_mir | 537 | hsa-miR-541-5p-479000_mir | 587 | hsa-miR-573-479055_mir |
| 438 | hsa-miR-452-5p-478109_mir | 488 | hsa-miR-513b-5p-479297_mir | 538 | hsa-miR-542-3p-478153_mir | 588 | hsa-miR-574-3p-478163_mir |
| 439 | hsa-miR-454-3p-478329_mir | 489 | hsa-miR-513c-5p-479349_mir | 539 | hsa-miR-542-5p-478337_mir | 589 | hsa-miR-575-479056_mir |
| 440 | hsa-miR-454-5p-478919_mir | 490 | hsa-miR-515-3p-478976_mir | 540 | hsa-miR-543-478155_mir | 590 | hsa-miR-576-3p-478164_mir |
| 441 | hsa-miR-455-3p-478112_mir | 491 | hsa-miR-515-5p-478147_mir | 541 | hsa-miR-544a-478156_mir | 591 | hsa-miR-576-5p-478165_mir |
| 442 | hsa-miR-455-5p-478113_mir | 492 | hsa-miR-516a-3p_hsa-miR-516b-3p-478977_mir | 542 | hsa-miR-545-3p-479002_mir | 592 | hsa-miR-577-479057_mir |
| 443 | hsa-miR-483-3p-478122_mir | 493 | hsa-miR-516a-5p-478978_mir | 543 | hsa-miR-545-5p-479003_mir | 593 | hsa-miR-578-479058_mir |
| 444 | hsa-miR-483-5p-478432_mir | 494 | hsa-miR-516b-5p-478979_mir | 544 | hsa-miR-548a-3p-478157_mir | 594 | hsa-miR-579-3p-479059_mir |
| 445 | hsa-miR-484-478308_mir | 495 | hsa-miR-517-5p-478980_mir | 545 | hsa-miR-548a-5p-479501_mir | 595 | hsa-miR-580-3p-479061_mir |
| 446 | hsa-miR-485-3p-478125_mir | 496 | hsa-miR-517a-3p_hsa-miR-517b-3p-479485_mir | 546 | hsa-miR-548am-5p_hsa-miR-548c-5p_hsa-miR-548o-5p-480872_mir | 596 | hsa-miR-581-479063_mir |
| 447 | hsa-miR-485-5p-478126_mir | 497 | hsa-miR-517c-3p-479487_mir | 547 | hsa-miR-548b-3p-479018_mir | 597 | hsa-miR-582-3p-479064_mir |
| 448 | hsa-miR-486-3p-478422_mir | 498 | hsa-miR-518a-3p-478981_mir | 548 | hsa-miR-548b-5p-478589_mir | 598 | hsa-miR-582-5p-478166_mir |
| 449 | hsa-miR-486-5p-478128_mir | 499 | hsa-miR-518a-5p_hsa-miR-527-479249_mir | 549 | hsa-miR-548c-3p-479537_mir | 599 | hsa-miR-583-479065_mir |
| 450 | hsa-miR-487a-3p-477826_mir | 500 | hsa-miR-518b-478148_mir | 550 | hsa-miR-548d-3p-477833_mir | 600 | hsa-miR-584-5p-478167_mir |

| 601 | hsa-miR-585-3p-479067_mir | 651 | hsa-miR-633-479115_mir | 701 | hsa-miR-769-5p-478203_mir | 751 | hsa-miR-99b-3p-478216_mir |
| --- | --- | --- | --- | --- | --- | --- | --- |
| 602 | hsa-miR-586-479069_mir | 652 | hsa-miR-634-479116_mir | 702 | hsa-miR-770-5p-479178_mir | 752 | hsa-miR-99b-5p-478343_mir |
| 603 | hsa-miR-587-479070_mir | 653 | hsa-miR-635-479117_mir | 703 | hsa-miR-802-479181_mir | | |
| 604 | hsa-miR-588-479071_mir | 654 | hsa-miR-636-478185_mir | 704 | hsa-miR-873-5p-478204_mir | | |
| 605 | hsa-miR-589-3p-479072_mir | 655 | hsa-miR-637-478186_mir | 705 | hsa-miR-874-3p-478205_mir | | |
| 606 | hsa-miR-589-5p-479073_mir | 656 | hsa-miR-638-478187_mir | 706 | hsa-miR-875-3p-479184_mir | | |
| 607 | hsa-miR-590-3p-478168_mir | 657 | hsa-miR-639-479118_mir | 707 | hsa-miR-875-5p-479185_mir | | |
| 608 | hsa-miR-590-5p-478367_mir | 658 | hsa-miR-640-479119_mir | 708 | hsa-miR-876-3p-479186_mir | | |
| 609 | hsa-miR-591-479074_mir | 659 | hsa-miR-641-479120_mir | 709 | hsa-miR-876-5p-479187_mir | | |
| 610 | hsa-miR-592-479075_mir | 660 | hsa-miR-642a-5p-479121_mir | 710 | hsa-miR-885-3p-479188_mir | | |
| 611 | hsa-miR-593-3p-479076_mir | 661 | hsa-miR-643-479123_mir | 711 | hsa-miR-885-5p-478207_mir | | |
| 612 | hsa-miR-593-5p-479077_mir | 662 | hsa-miR-644a-479124_mir | 712 | hsa-miR-887-3p-479189_mir | | |
| 613 | hsa-miR-595-479078_mir | 663 | hsa-miR-645-478188_mir | 713 | hsa-miR-888-3p-479191_mir | | |
| 614 | hsa-miR-596-478338_mir | 664 | hsa-miR-646-479125_mir | 714 | hsa-miR-888-5p-479192_mir | | |
| 615 | hsa-miR-597-5p-478339_mir | 665 | hsa-miR-647-479126_mir | 715 | hsa-miR-889-3p-478208_mir | | |
| 616 | hsa-miR-598-3p-478172_mir | 666 | hsa-miR-648-479127_mir | 716 | hsa-miR-890-479194_mir | | |
| 617 | hsa-miR-599-479081_mir | 667 | hsa-miR-649-479128_mir | 717 | hsa-miR-891a-5p-479196_mir | | |
| 618 | hsa-miR-600-479082_mir | 668 | hsa-miR-650-479129_mir | 718 | hsa-miR-891b-479197_mir | | |
| 619 | hsa-miR-601-478173_mir | 669 | hsa-miR-651-5p-479131_mir | 719 | hsa-miR-892a-479371_mir | | |
| 620 | hsa-miR-603-479084_mir | 670 | hsa-miR-652-3p-478189_mir | 720 | hsa-miR-892b-479198_mir | | |
| 621 | hsa-miR-604-479085_mir | 671 | hsa-miR-653-5p-479134_mir | 721 | hsa-miR-9-3p-478211_mir | | |
| 622 | hsa-miR-605-5p-478174_mir | 672 | hsa-miR-654-3p-479135_mir | 722 | hsa-miR-9-5p-478214_mir | | |
| 623 | hsa-miR-606-479087_mir | 673 | hsa-miR-654-5p-478368_mir | 723 | hsa-miR-920-479201_mir | | |
| 624 | hsa-miR-607-479088_mir | 674 | hsa-miR-655-3p-478191_mir | 724 | hsa-miR-921-479202_mir | | |
| 625 | hsa-miR-608-479089_mir | 675 | hsa-miR-656-3p-479137_mir | 725 | hsa-miR-922-479203_mir | | |
| 626 | hsa-miR-609-479091_mir | 676 | hsa-miR-657-479139_mir | 726 | hsa-miR-924-479204_mir | | |
| 627 | hsa-miR-613-479097_mir | 677 | hsa-miR-658-479140_mir | 727 | hsa-miR-92a-1-5p-479205_mir | | |
| 628 | hsa-miR-614-479098_mir | 678 | hsa-miR-659-3p-479141_mir | 728 | hsa-miR-92a-2-5p-479206_mir | | |
| 629 | hsa-miR-615-3p-478175_mir | 679 | hsa-miR-660-5p-478192_mir | 729 | hsa-miR-92a-3p-477827_mir | | |
| 630 | hsa-miR-615-5p-478176_mir | 680 | hsa-miR-661-479144_mir | 730 | hsa-miR-92b-3p-477823_mir | | |
| 631 | hsa-miR-616-3p-478177_mir | 681 | hsa-miR-662-479145_mir | 731 | hsa-miR-92b-5p-479207_mir | | |
| 632 | hsa-miR-616-5p-479099_mir | 682 | hsa-miR-663b-479146_mir | 732 | hsa-miR-93-3p-478209_mir | | |
| 633 | hsa-miR-617-479100_mir | 683 | hsa-miR-664a-3p-478193_mir | 733 | hsa-miR-93-5p-478210_mir | | |
| 634 | hsa-miR-618-479101_mir | 684 | hsa-miR-665-479150_mir | 734 | hsa-miR-933-479208_mir | | |
| 635 | hsa-miR-620-479104_mir | 685 | hsa-miR-668-3p-479151_mir | 735 | hsa-miR-934-479209_mir | | |
| 636 | hsa-miR-621-479105_mir | 686 | hsa-miR-671-3p-478194_mir | 736 | hsa-miR-935-479210_mir | | |
| 637 | hsa-miR-622-479106_mir | 687 | hsa-miR-675-5p-478196_mir | 737 | hsa-miR-936-479211_mir | | |
| 638 | hsa-miR-623-479512_mir | 688 | hsa-miR-7-1-3p-478198_mir | 738 | hsa-miR-937-3p-479212_mir | | |
| 639 | hsa-miR-624-3p-479108_mir | 689 | hsa-miR-7-2-3p-478199_mir | 739 | hsa-miR-938-479214_mir | | |
| 640 | hsa-miR-624-5p-478178_mir | 690 | hsa-miR-7-5p-478341_mir | 740 | hsa-miR-939-5p-478245_mir | | |
| 641 | hsa-miR-625-3p-478179_mir | 691 | hsa-miR-708-3p-479162_mir | 741 | hsa-miR-941-479217_mir | | |
| 642 | hsa-miR-625-5p-479469_mir | 692 | hsa-miR-708-5p-478197_mir | 742 | hsa-miR-942-5p-478212_mir | | |
| 643 | hsa-miR-626-479110_mir | 693 | hsa-miR-744-3p-479165_mir | 743 | hsa-miR-943-479219_mir | | |
| 644 | hsa-miR-627-5p-478427_mir | 694 | hsa-miR-744-5p-478200_mir | 744 | hsa-miR-944-479220_mir | | |
| 645 | hsa-miR-628-3p-478181_mir | 695 | hsa-miR-758-3p-479166_mir | 745 | hsa-miR-95-3p-478213_mir | | |
| 646 | hsa-miR-628-5p-479112_mir | 696 | hsa-miR-765-479173_mir | 746 | hsa-miR-96-3p-479222_mir | | |
| 647 | hsa-miR-629-3p-478182_mir | 697 | hsa-miR-766-3p-478342_mir | 747 | hsa-miR-96-5p-478215_mir | | |
| 648 | hsa-miR-629-5p-478183_mir | 698 | hsa-miR-767-3p-479175_mir | 748 | hsa-miR-98-5p-478590_mir | | |
| 649 | hsa-miR-630-479113_mir | 699 | hsa-miR-767-5p-479176_mir | 749 | hsa-miR-99a-3p-479224_mir | | |
| 650 | hsa-miR-631-478340_mir | 700 | hsa-miR-769-3p-479177_mir | 750 | hsa-miR-99a-5p-478519_mir | | |
