# Supplementary figures and images for: Osteoclast-like stromal giant cells in breast cancer likely belong to the spectrum of immunosuppressive tumor-associated macrophages
Source: Front Mol Biosci. 2022 Aug 26;9:894247. doi: 10.3389/fmolb.2022.894247 (PMC9462457; doi:10.3389/fmolb.2022.894247)

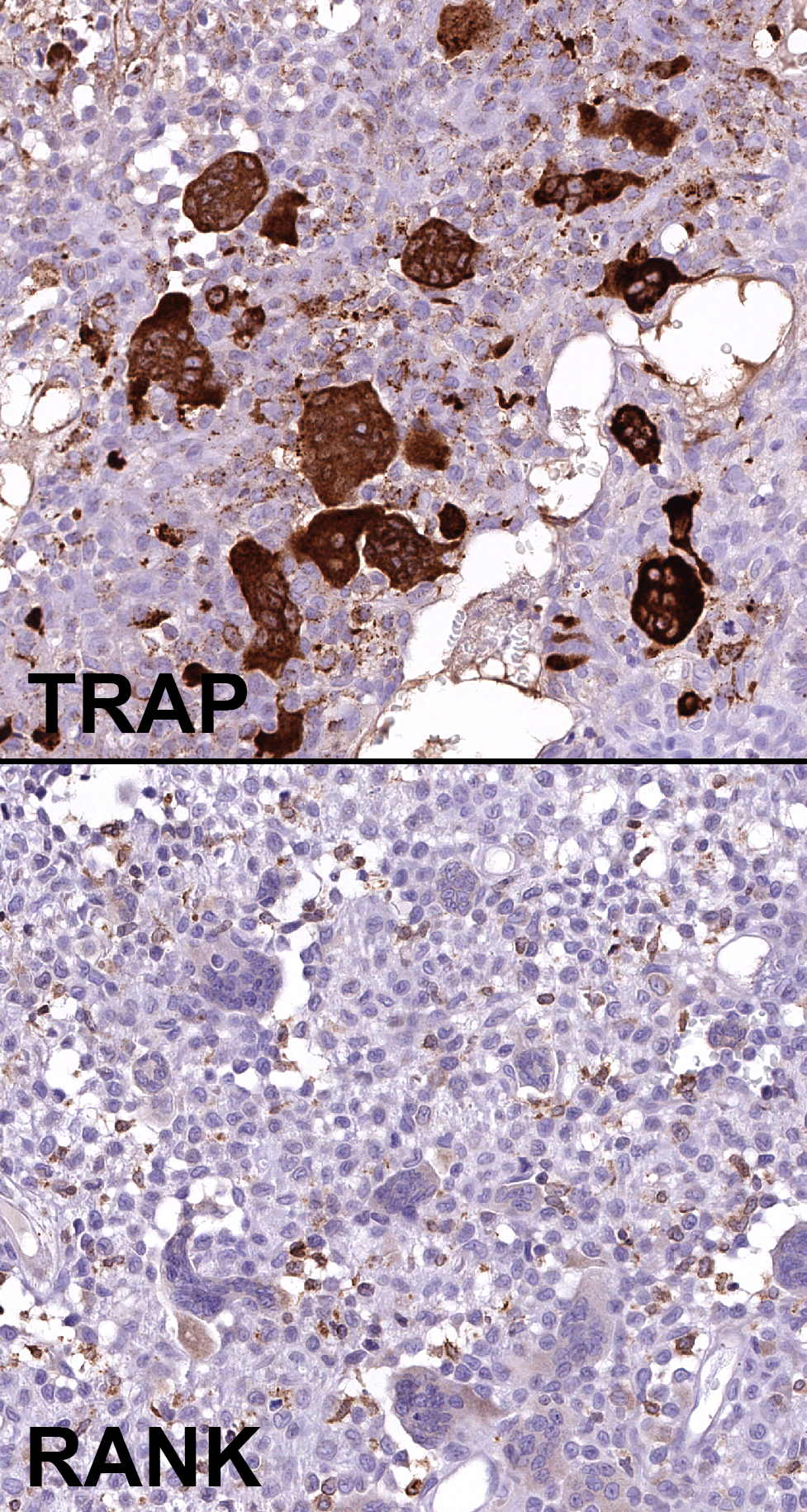

Supplement: Supplementary file 2 [file Image1.TIF]
