# Supplementary material for: Osteoclast-like stromal giant cells in breast cancer likely belong to the spectrum of immunosuppressive tumor-associated macrophages
Source: Front Mol Biosci. 2022 Aug 26;9:894247. doi: 10.3389/fmolb.2022.894247 (PMC9462457; doi:10.3389/fmolb.2022.894247)
Supplement: Supplementary file 3 [file Table2.DOCX]

| **Supplementary Table s2.** List of miRNA’s with cycle threshold less than 35. | | | | | |
| --- | --- | --- | --- | --- | --- |
|  |  |  |  |  |  |
| 1 | hsa-miR-181a-5p | 51 | hsa-miR-15a-5p | 101 | hsa-miR-126-5p |
| 2 | hsa-miR-143-3p | 52 | hsa-miR-210-3p | 102 | hsa-miR-652-3p |
| 3 | hsa-miR-181b-5p | 53 | hsa-let-7d-5p | 103 | hsa-miR-10a-5p |
| 4 | hsa-let-7b-5p | 54 | hsa-miR-132-3p | 104 | hsa-miR-424-5p |
| 5 | hsa-miR-195-5p | 55 | hsa-miR-22-5p | 105 | hsa-let-7i-5p |
| 6 | hsa-miR-181c-5p | 56 | hsa-miR-486-5p | 106 | hsa-miR-101-3p |
| 7 | hsa-miR-222-3p | 57 | hsa-miR-302d-3p | 107 | hsa-miR-99b-5p |
| 8 | hsa-miR-455-5p | 58 | hsa-miR-125a-3p | 108 | hsa-miR-140-3p |
| 9 | hsa-miR-181a-3p | 59 | hsa-miR-194-5p | 109 | hsa-miR-19b-3p |
| 10 | hsa-let-7i-3p | 60 | hsa-miR-124-3p | 110 | hsa-miR-324-5p |
| 11 | hsa-miR-130b-3p | 61 | hsa-miR-99a-5p | 111 | hsa-miR-151a-5p |
| 12 | hsa-let-7b-3p | 62 | hsa-miR-148b-3p | 112 | hsa-miR-30a-3p |
| 13 | hsa-miR-190a-5p | 63 | hsa-miR-660-5p | 113 | hsa-miR-28-3p |
| 14 | hsa-miR-142-5p | 64 | hsa-miR-574-3p | 114 | hsa-miR-423-3p |
| 15 | hsa-miR-301a-3p | 65 | hsa-let-7e-5p | 115 | hsa-miR-193a-5p |
| 16 | hsa-miR-941 | 66 | hsa-miR-423-5p | 116 | hsa-miR-185-5p |
| 17 | hsa-miR-21-3p | 67 | hsa-miR-455-3p | 117 | hsa-miR-1301-3p |
| 18 | hsa-miR-26b-5p | 68 | hsa-miR-19a-3p | 118 | hsa-miR-328-3p |
| 19 | hsa-miR-221-3p | 69 | hsa-miR-708-5p | 119 | hsa-miR-196a-5p |
| 20 | hsa-miR-125a-5p | 70 | hsa-miR-331-3p | 120 | hsa-miR-585-3p |
| 21 | hsa-miR-214-3p | 71 | hsa-miR-1260a | 121 | hsa-miR-128-3p |
| 22 | hsa-miR-155-5p | 72 | hsa-miR-193a-3p | 122 | hsa-miR-615-3p |
| 23 | hsa-miR-10b-5p | 73 | hsa-miR-450a-5p | 123 | hsa-miR-362-3p |
| 24 | hsa-miR-361-5p | 74 | hsa-miR-505-3p | 124 | hsa-miR-199a-3p |
| 25 | hsa-miR-376a-3p | 75 | hsa-miR-524-3p | 125 | hsa-miR-126-3p |
| 26 | hsa-miR-32-5p | 76 | hsa-miR-374b-5p | 126 | hsa-miR-148a-3p |
| 27 | hsa-miR-100-5p | 77 | hsa-miR-1-3p | 127 | hsa-miR-193b-3p |
| 28 | hsa-miR-31-3p | 78 | hsa-miR-146b-5p | 128 | hsa-miR-30c-5p |
| 29 | hsa-miR-874-3p | 79 | hsa-miR-20b-5p | 129 | hsa-miR-223-3p |
| 30 | hsa-miR-140-5p | 80 | hsa-miR-99a-3p | 130 | hsa-miR-29a-3p |
| 31 | hsa-miR-145-5p | 81 | hsa-miR-29c-3p |  |  |
| 32 | hsa-miR-451a- | 82 | hsa-let-7d-3p |  |  |
| 33 | hsa-miR-103a-3p | 83 | hsa-miR-151a-3p |  |  |
| 34 | hsa-miR-28-5p | 84 | hsa-miR-342-3p |  |  |
| 35 | hsa-miR-378a-3p | 85 | hsa-miR-130a-3p |  |  |
| 36 | hsa-miR-197-3p | 86 | hsa-miR-106b-5p |  |  |
| 37 | hsa-miR-1296-5p | 87 | hsa-miR-340-5p |  |  |
| 38 | hsa-let-7g-5p | 88 | hsa-miR-484 |  |  |
| 39 | hsa-miR-218-5p | 89 | hsa-miR-361-3p |  |  |
| 40 | hsa-miR-92b-3p | 90 | hsa-miR-205-5p |  |  |
| 41 | hsa-miR-150-5p | 91 | hsa-miR-503-5p |  |  |
| 42 | hsa-miR-365a-3p | 92 | hsa-miR-24-3p |  |  |
| 43 | hsa-miR-17-5p | 93 | hsa-miR-21-5p |  |  |
| 44 | hsa-miR-199a-5p | 94 | hsa-miR-26a-5p |  |  |
| 45 | hsa-miR-362-5p | 95 | hsa-miR-22-3p |  |  |
| 46 | hsa-miR-151b | 96 | hsa-miR-16-5p |  |  |
| 47 | hsa-miR-92a-3p | 97 | hsa-miR-23a-3p |  |  |
| 48 | hsa-let-7f-5p | 98 | hsa-miR-152-3p |  |  |
| 49 | hsa-miR-146a-5p | 99 | hsa-miR-20a-5p |  |  |
| 50 | hsa-miR-27b-3p | 100 | hsa-miR-25-3p |  |  |
